# Sirt7 promotes adipogenesis by binding to and inhibiting Sirt1

**DOI:** 10.1186/1753-6561-6-S3-P57

**Published:** 2012-06-01

**Authors:** Eva Bober, Jian Fang, Christian Smolka, Alessandro Ianni, Olesya Vakhrusheva, Marcus Krüger, Thomas Braun

**Affiliations:** 1 Max Planck Institute for Heart and Lung Research, D-61321 Bad Nauheim, Germany

## Background

Members of the mammalian sirtuin family, Sirt1 – Sirt7, are known to regulate metabolic processes especially carbohydrate and fat metabolism [1,2]. Sirt1 and Sirt2 inhibit adipocyte differentiation [3,4] while Sirt1 and Sirt6 prevent liver steatosis [5]. These examples illustrate a synergistic action of different sirtuins in promoting lean, “healthy” phenotypes. We have previously shown that Sirt7 knockout mice display signs of premature aging, suffer from progressive cardiomyopathy and have a reduced lifespan [6]. Here, we investigate the biological function of Sirt7 in the regulation of metabolism in white adipose tissue (WAT) and liver.

## Results

To discover new regulators of Sirt1 activity we performed an unbiased screen for molecules that might interact with Sirt1 using a label free quantitative mass spectrometry based co-immunoprecipitation strategy. We identified Sirt7 as a novel Sirt1 binding protein. The interaction between Sirt1 and Sirt7 was confirmed by immunoprecipitation of endogenous proteins and GST pull-down assays. Sirt1 protein expression and enzymatic activity was increased in WAT of Sirt7 knockout mice leading to age-dependent lipodystrophic phenotype. Increased Sirt1 activity might account for resistance of Sirt7 knockout mice fed high fat diet against liver steatosis. In vitro experiments demonstrated a diminished ability of Sirt7 deficient MEFs and primary preadipocytes to undergo adipogenesis. These defects were rescued by knock-down of Sirt1 or in cells deficient for one Sirt1 allele (Sirt1+/-; Sirt7-/-).

## Conclusions

Our results highlight the importance of cross-regulatory circuits among individual members of the sirtuin family in organismal homeostasis. Lack of Sirt7 leads to a sustained activation of Sirt1. Apparently, such un-physiologically exaggerated, persistent Sirt1 activation results in metabolic dysfunction and nullifies its principally beneficial effects such as fat mobilization and inhibition of adipogenesis.
